# Evaluation of Diastolic Dysfunction in Pituitary Adenomas: A Comparative Single-Center Study Among Acromegaly, Prolactinoma, and Nonfunctioning Pituitary Adenomas

**DOI:** 10.3390/medicina62101859

**Published:** 2026-09-25

**Authors:** Rabia Eker Akıllı, Seher Çetinkaya Altuntaş, Omer Tepe, Anıl Akray, Ülcaz Perihan Aksoydan Zubaroglu, Abdulkadir İltaş, Tugay Erdem, Eylül Akbal, Emre Çağlar Çağlayan

**Affiliations:** 1 Department of Cardiology, Faculty of Medicine, Cukurova University, 01330 Adana, Turkey; 2 Department of Endocrinology and Metabolic Diseases, University of Health Sciences, Bursa High Specialization Training and Research Hospital, 16310 Bursa, Turkey; 3 Department of Cardiology, Education and Research Hospital, 80010 Osmaniye, Turkey; 4 Department of Cardiology, Iskenderun State Hospital, 31240 Hatay, Turkey; 5 Department of Endocrinology, Faculty of Medicine, Mersin University, 33343 Mersin, Turkey; 6 Department of Cardiology, Omer Halisdemir University, Education and Research Hospital, 51100 Nigde, Turkey; 7 Department of Radiology, Faculty of Medicine, Çukurova University, 01330 Adana, Turkey

**Keywords:** pituitary adenoma, acromegaly, prolactinoma, left ventricular diastolic dysfunction, echocardiography, doppler, carotid intima-media thickness

## Abstract

*Background and Objectives:* Pituitary adenomas may be associated with metabolic and cardiovascular abnormalities, but comparative evidence across subtypes is limited. We compared cardiometabolic and echocardiographic profiles, left ventricular diastolic dysfunction (LVDD), and carotid intima–media thickness (CIMT) among patients with prolactinoma, acromegaly, or nonfunctioning pituitary adenoma (NFPA) and healthy controls and investigated factors associated with LVDD. *Materials and Methods:* This cross-sectional comparative study included 214 participants evaluated between 2016 and 2018: 91 with prolactinoma, 26 with acromegaly, 45 with NFPA, and 52 healthy controls. Participants underwent clinical, biochemical, echocardiographic, and CIMT assessment. LVDD was diagnosed and graded according to the 2016 American Society of Echocardiography (ASE) and European Association of Cardiovascular Imaging (EACVI) recommendations. Factors associated with LVDD were evaluated using multivariable logistic regression. *Results:* The mean age was 41.3 ± 12.4 years, and 64.5% of participants were women. Acromegaly was associated with the highest fasting glucose, HbA1c, insulin, and homeostasis model assessment of insulin resistance (HOMA-IR) values (all *p* < 0.001) and, compared with controls, a higher left atrial volume index (LAVI) and average E/e′ ratio and lower left ventricular ejection fraction (despite preserved ejection fraction), E/A ratio, and lateral e′ velocity. CIMT was higher in NFPA than in prolactinoma. LVDD was present in 21.5% of the cohort and in 5.8%, 19.8%, 46.2%, and 28.9% of the control, prolactinoma, acromegaly, and NFPA groups, respectively (*p* < 0.001); 84.8% of cases were grade I. Increasing age was independently associated with LVDD (OR 1.265 per year, 95% CI 1.155–1.385; *p* < 0.001), and diagnostic group was independently associated with LVDD overall (*p* = 0.004). Compared with controls, adjusted LVDD odds were higher in prolactinoma (OR 10.9, 95% CI 1.34–89.68), acromegaly (OR 167.5, 95% CI 10.3–2721.4), and NFPA (OR 12.0, 95% CI 1.4–104.8). *Conclusions:* Pituitary adenoma subtypes were associated with distinct cardiometabolic and echocardiographic profiles and a higher prevalence of LVDD than in healthy controls, particularly in acromegaly. Most LVDD cases were grade I, indicating predominantly impaired relaxation. Although age and diagnostic group were associated with LVDD in the adjusted analysis, the wide confidence intervals warrant cautious interpretation and validation in larger cohorts.

## 1. Introduction

Pituitary adenomas are common intracranial neoplasms that form a clinically heterogeneous group, with manifestations determined by hormone secretion, tumor size, and associated pituitary dysfunction [1]. Their relevance extends beyond local mass effects and endocrine symptoms because hormone excess and pituitary deficiencies may be accompanied by metabolic and cardiovascular abnormalities. The nature and extent of these abnormalities may differ across adenoma subtypes [2].

Acromegaly results from sustained growth hormone (GH) and insulin-like growth factor-1 (IGF-1) excess and has the most extensively described cardiovascular phenotype among pituitary disorders [3]. Reported findings include increased left ventricular wall thickness, concentric remodeling, impaired ventricular relaxation, and, less commonly, clinically apparent systolic dysfunction. These changes are often considered part of acromegaly-related cardiomyopathy, although their expression may vary with disease duration, biochemical activity, age, blood pressure, and metabolic comorbidities [3,4]. Cardiac structure and function may improve after biochemical control, but recovery is not uniform.

The cardiovascular implications of prolactinoma are less clearly established. Hyperprolactinemia has been associated with insulin resistance, adverse lipid profiles, and impaired endothelial function, although available findings are not entirely consistent [5]. Echocardiographic studies have also suggested subtle abnormalities in left ventricular longitudinal function or diastolic relaxation in some patients without overt cardiovascular disease [6,7]. The clinical relevance and reversibility of these observations remain uncertain.

Nonfunctioning pituitary adenomas (NFPAs) lack clinically relevant hormone hypersecretion but may be accompanied by hypopituitarism, metabolic comorbidities, or treatment-related factors that could influence cardiovascular status [8]. Their cardiac phenotype has received less attention than that of functioning adenomas, and it remains uncertain whether reported abnormalities primarily reflect pituitary deficiencies, conventional cardiovascular risk factors, or other disease-related characteristics.

Left ventricular diastolic dysfunction may serve as an echocardiographic marker of myocardial involvement before systolic impairment becomes evident. Transmitral flow, mitral annular tissue Doppler velocities, the average E/e′ ratio, and LAVI provide complementary information on ventricular relaxation and filling pressure. Diastolic abnormalities have been reported separately in acromegaly and chronic hyperprolactinemia [7,9], but comparative studies including different adenoma subtypes and healthy controls remain limited.

This study aimed to compare clinical and metabolic characteristics, cardiac structure and function, the prevalence and grade of LVDD, and CIMT among patients with acromegaly, prolactinoma, or NFPA and healthy controls. We also investigated clinical and echocardiographic factors independently associated with LVDD.

## 2. Materials and Methods

### 2.1. Study Design and Participants

This retrospective, cross-sectional comparative study included 214 participants evaluated between 2016 and 2018. The patient groups comprised individuals with prolactinoma, acromegaly, or NFPA who attended the endocrinology and cardiology outpatient clinics of our institution. Apparently healthy individuals constituted the control group. Clinical data for all participants, including those in both the patient and control groups, were obtained from existing institutional hospital records. No a priori sample-size calculation was performed; the sample consisted of eligible participants available during the predefined study period.

Pituitary adenomas were diagnosed on the basis of clinical, biochemical, and pituitary magnetic resonance imaging (MRI) findings. Acromegaly was diagnosed based on an elevated age-adjusted serum IGF-1 concentration and failure of serum GH to suppress to <1 ng/mL during a 75-g oral glucose tolerance test, in accordance with the diagnostic criteria applicable during the study period. Standardized IGF-1 values expressed as multiples of the upper limit of normal were not consistently available in the retrospective records; therefore, the >1.3 × upper-limit-of-normal threshold could not be applied retrospectively. Prolactinoma was diagnosed in the presence of persistent hyperprolactinemia and a compatible pituitary lesion on MRI after exclusion of physiological, pharmacological, and other secondary causes of hyperprolactinemia. The laboratory thresholds used to define hyperprolactinemia were >25 ng/mL in women and >20 ng/mL in men. NFPA was defined by radiological evidence of a pituitary adenoma without clinical or biochemical evidence of hormone hypersecretion.

Exclusion criteria included diabetes insipidus and concomitant endocrine disorders unrelated to the underlying pituitary adenoma, such as untreated or uncontrolled overt thyroid dysfunction. ACTH- and TSH-secreting pituitary adenomas were not included because their limited numbers precluded meaningful subgroup analyses. Individuals with hypertension, diabetes mellitus, or clinically significant cardiovascular disease were excluded to reduce potential confounding related to conditions that contribute to left ventricular diastolic dysfunction. Clinically significant cardiovascular disease was defined as obstructive coronary artery disease, previous myocardial infarction or coronary revascularization, inducible myocardial ischemia, regional wall-motion abnormalities, heart failure, cardiomyopathy, congenital heart disease, significant valvular heart disease, or arrhythmias that could interfere with Doppler measurements. Additional exclusion criteria included a history of stroke, transient ischemic attack, or pulmonary embolism; severe chronic pulmonary disease or respiratory failure; advanced chronic kidney or liver disease; active infection; systemic autoimmune or inflammatory disease; active malignancy; and pregnancy. Participants with echocardiographic image quality insufficient for reliable assessment of cardiac structure, diastolic function, or tissue Doppler parameters were also excluded.

### 2.2. Clinical and Biochemical Assessment

Demographic characteristics, medical history, pituitary surgical history, hormone-replacement therapy, and the presence of pituitary insufficiency were recorded. Anthropometric assessment included height, body weight, body mass index (BMI), and waist circumference. BMI was calculated as body weight in kilograms divided by height in meters squared (kg/m^2^).

Venous blood samples were obtained after an overnight fast. Fasting blood glucose, glycated hemoglobin (HbA1c), total cholesterol, low-density lipoprotein (LDL) cholesterol, high-density lipoprotein (HDL) cholesterol, triglycerides (TG), fasting insulin, and mean platelet volume (MPV) were recorded. The homeostasis model assessment of insulin resistance (HOMA-IR) was calculated as follows:

HOMA-IR = fasting insulin (µIU/mL) × fasting blood glucose (mg/dL)/405

Hormonal assessment included serum GH, IGF-1, prolactin, and other anterior pituitary hormones, as clinically indicated. To minimize inter-assay variability, all biochemical and hormonal analyses were performed in the same institutional laboratory after overnight fasting using standard enzymatic and immunoassay methods by cobas e 601 (Roche Diagnostics GmbH, Mannheim, Germany).

### 2.3. Echocardiographic Assessment

All participants underwent comprehensive transthoracic echocardiography using a Vivid E95 ultrasound system (GE Vingmed Ultrasound AS, Horten, Norway). Examinations were performed by an experienced cardiologist who was blinded to the participants’ clinical and biochemical data. Standard two-dimensional (2D), pulsed-wave Doppler (PWD), and tissue Doppler imaging (TDI) measurements were obtained during the study period in accordance with the 2016 recommendations of ASE and EACVI) [10].

Left atrial (LA) diameter, interventricular septal (IVS) thickness, left ventricular posterior wall (LVPW) thickness, left ventricular end-diastolic diameter (LVEDD), and left ventricular end-systolic diameter (LVESD) were measured using standard techniques. Left ventricular ejection fraction (LVEF) was calculated using the modified biplane Simpson method. Left atrial volume was measured using the biplane area–length method and indexed to body surface area to derive LAVI (mL/m^2^).

Transmitral early (E) and late (A) diastolic velocities, the E/A ratio, and mitral E-wave deceleration time (DT) were measured using pulsed-wave Doppler. Tissue Doppler imaging was used to measure early diastolic (e′), late diastolic (a′), and systolic (s′) myocardial velocities at the septal and lateral mitral annuli and the lateral tricuspid annulus. Isovolumic relaxation time (IVRT), isovolumic contraction time (IVCT), and ejection time (ET) were also recorded at the septal, lateral, and right ventricular annular sites. Average e′ velocity was calculated as the arithmetic mean of the septal and lateral e′ velocities, and the average E/e′ ratio was defined as the transmitral E-wave velocity divided by the average e′ velocity. Right heart dimensions (RA/RV) and tricuspid annular plane systolic excursion (TAPSE) were measured using standard echocardiographic views.

*Assessment of Left Ventricular Diastolic Function*; left ventricular diastolic function (LVDD) was evaluated using transmitral early (E) and late (A) diastolic flow velocities, the E/A ratio, septal and lateral early diastolic mitral annular velocities (e′), the E/e′ ratio, peak tricuspid regurgitation velocity (TRV), and LAVI. In participants with preserved left ventricular ejection fraction and no known myocardial disease, LVDD was identified using the four variables recommended by the 2016 ASE/EACVI algorithm: septal e′ velocity < 7 cm/s or lateral e′ velocity < 10 cm/s, average E/e′ ratio > 14, peak TRV > 2.8 m/s, and LAVI > 34 mL/m^2^. Diastolic function was classified as abnormal when more than 50% of the available variables exceeded their respective cutoff values, normal when fewer than 50% were abnormal, and indeterminate when exactly 50% were abnormal. Diastolic dysfunction was subsequently graded according to transmitral flow and estimated left ventricular filling pressure. An E/A ratio ≤ 0.8 accompanied by a peak E velocity ≤ 50 cm/s was classified as grade I diastolic dysfunction, whereas an E/A ratio ≥2.0 was classified as grade III diastolic dysfunction. When the E/A ratio was ≤0.8 with a peak E velocity > 50 cm/s or was between 0.8 and 2.0, the average E/e′ ratio, peak TRV, and LAVI were considered together. At least two abnormal parameters indicated elevated filling pressure and grade II diastolic dysfunction, whereas at least two normal parameters indicated normal filling pressure and grade I diastolic dysfunction.

Carotid intima–media thickness (CIMT) was assessed during the same examination session using the same ultrasound system equipped with a 9L-D wideband linear-array transducer with a frequency range of 2.4–10.0 MHz. Participants were examined in the supine position with the neck mildly extended and the head rotated away from the side being evaluated. Both common carotid arteries were visualized in the longitudinal plane using high-resolution B-mode ultrasonography. In accordance with the Mannheim consensus, CIMT was defined as the distance between the leading edge of the lumen–intima interface and the leading edge of the media–adventitia interface [11]. Measurements were obtained at end-diastole from a plaque-free segment of the far wall of the distal common carotid artery, within approximately 10 mm proximal to the carotid bulb. Mean CIMT values were calculated separately for the right and left common carotid arteries, and the arithmetic mean of the two sides was used as the participant-level CIMT value in the statistical analyses.

### 2.4. Statistical Analysis

Statistical analyses were performed using IBM SPSS Statistics, version 20.0 (IBM Corp., Armonk, NY, USA). The distribution of continuous variables was evaluated using the Kolmogorov–Smirnov test together with visual inspection of distribution plots, if performed. Normally distributed continuous variables were summarized as mean ± standard deviation, whereas nonnormally distributed variables were summarized as median (Q1–Q3). Comparisons of continuous variables across the four study groups were performed using one-way analysis of variance for normally distributed data and the Kruskal–Wallis test for nonnormally distributed data. When an overall between-group difference was significant, Bonferroni or Dunn pairwise post hoc comparisons were performed. Categorical variables were compared using the Pearson chi-square test or Fisher exact test, as appropriate. The prevalence of left ventricular diastolic dysfunction was compared across the four groups using the chi-square test. A Logistic regression was used to examine factors associated with left ventricular diastolic dysfunction. Candidate variables were selected on the basis of clinical relevance and/or an association in univariable analyses (*p* < 0.05). Multicollinearity was assessed before construction of the multivariable model. Smoking status was not included in the multivariable model because the number of smokers was too small to support reliable estimation of an adjusted association with LVDD. The final model of logistic regretion analyses included age, BMI, LDL, HbA1c, LVEDD, LVPW thickness, RV diame-ter, and diagnostic group. Results were expressed as odds ratios with 95% confidence intervals. All tests were two-sided, and *p* < 0.05 was considered statistically significant.

### 2.5. Ethical Considerations

The study protocol was approved by the Çukurova University Faculty of Medicine Ethics Committee with decision number 107/12 on 22 January 2021. Written informed consent was not obtained for this retrospective study, which used existing hospital records. The study was conducted in accordance with the principles of the Declaration of Helsinki.

## 3. Results

The study consisted of 76 males (35.5%) and 138 females (64.5%) with mean age 41.3 ± 12.4 years). Participants were categorized into four groups based on pituitary adenoma subtype: prolactinoma (*n* = 91, 42.6%), non-functional pituitary adenoma (NFPA; *n* = 45, 21.0%), acromegaly (*n* = 26, 12.1%), and healthy controls (*n* = 52, 24.3%).

Although age did not differ significantly among the groups (*p* = 0.120), significant between-group differences were observed in BMI (*p* = 0.001), height (*p* = 0.001), body weight (*p* < 0.001), and waist circumference (*p* < 0.001). BMI was higher in the acromegaly and prolactinoma groups than in the control group, whereas height was greater in the NFPA and acromegaly groups than in the control group. All patient groups had higher body weight and waist circumference than the control group. Among the patient groups, body weight (*p* = 0.023) and waist circumference (*p* = 0.026) were higher in the acromegaly group than in the prolactinoma group. Tumor size also differed significantly among the patient groups (*p* = 0.013). The largest tumors were observed in the acromegaly 10 (7–12 mm) followed by the NFPA group 9 (5–20.2 mm) and the prolactinoma 6 (3–10 mm) groups (Table 1).

Regarding biochemical and metabolic parameters, no significant between-group differences were observed in HDL (*p* = 0.307), total cholesterol (*p* = 0.118), or TG levels (*p* = 0.051). In contrast, fasting blood glucose (*p* < 0.001), HbA1c (*p* < 0.001), LDL (*p* = 0.003), insulin (*p* < 0.001), HOMA-IR (*p* < 0.001), and MPV (*p* < 0.001) differed significantly among the groups. The highest fasting blood glucose, HbA1c, insulin, and HOMA-IR values were observed in the acromegaly group. Insulin and HOMA-IR values were also higher in the prolactinoma group than in the control group. LDL was highest in the prolactinoma group, whereas MPV was lower in the acromegaly group than in all other groups (Table 2).

No significant between-group differences were observed in LA diameter (*p* = 0.100), IVS thickness (*p* = 0.107), RV diameter (*p* = 0.062), mitral A-wave velocity (*p* = 0.252), DT (*p* = 0.480), tricuspid E-wave velocity (*p* = 0.185), TAPSE (*p* = 0.572), lateral s′ velocity (*p* = 0.090), septal e′ velocity (*p* = 0.053), TRV (*p* = 0.157) and RV TDI parameters (RV e′, a′, and s′ velocities, RV IVRT, IVCT, and ET).

In contrast, significant differences among the groups were observed in aortic diameter (*p* = 0.033), LAVI (*p* = 0.017), LVEF (*p* = 0.004), LVEDD (*p* = 0.010), LVESD (*p* = 0.001), LVPW thickness (*p* = 0.026), RA diameter (*p* = 0.006), mitral E-wave velocity (*p* < 0.001), mitral E/A ratio (*p* < 0.001), average E/e’ ratio (*p* = 0.001), tricuspid A-wave velocity (*p* = 0.026), lateral e′ velocity (*p* < 0.001), lateral a′ velocity (*p* = 0.005), lateral IVRT (*p* = 0.008), lateral IVCT (*p* = 0.017), septal a′ velocity (*p* < 0.001), septal s′ velocity (*p* = 0.025), septal IVRT (*p* = 0.032), septal IVCT (*p* = 0.026), average e′ velocity (*p* < 0.001), and CIMT (*p* = 0.026).

Aortic diameter (*p* = 0.044) was higher in the acromegaly group than in the control group. LAVI differed significantly among the study groups (*p* < 0.001). LAVI was higher in the acromegaly group than in the control (*p* = 0.003), prolactinoma (*p* = 0.003), and NFPA (*p* < 0.001) groups, whereas no significant pairwise differences were observed among the remaining groups (all *p* > 0.05).

Among the left ventricular systolic parameters, LVEF was lower in the acromegaly group than in the control group (*p* = 0.002), whereas LVESD was higher in both the NFPA (*p* = 0.039) and acromegaly (*p* = 0.001) groups than in the control group. LVPW was thicker in the acromegaly group than in the prolactinoma group (*p* = 0.021). RA diameter was also greater in the acromegaly group than in both the NFPA (*p* = 0.009) and prolactinoma (*p* = 0.035) groups.

Evaluation of left ventricular diastolic function and TDI parameters showed that mitral E-wave velocity was lower in the acromegaly group than in the other groups (all *p* < 0.05). The mitral E/A ratio was lower in the acromegaly group than in the control (*p* < 0.001) and prolactinoma (*p* = 0.002) groups and was also lower in the NFPA group than in the control group (*p* = 0.019). The average E/e′ ratio differed significantly among the groups; with the highest median value observed in the acromegaly group (*p* = 0.001). Lateral e′ velocity was lower in the acromegaly group than in all other groups (all *p* < 0.001) and was also lower in the prolactinoma group than in the control group (*p* = 0.009). Lateral IVRT was longer in the acromegaly group than in the prolactinoma group (*p* = 0.005). Septal a′ velocity was higher in all patient groups than in the control group (all *p* < 0.05), whereas septal s′ velocity was higher in the acromegaly group than in the control group (*p* = 0.048). Finally, CIMT was higher in the NFPA group than in the prolactinoma group (*p* = 0.026).

LVDD was found in 46 patients (21.5%) of the total cohort. When the LVDD stages of these individuals were evaluated on the basis of groups, all three patients in the control group were found to be in stage 1, while in the prolactinoma group, 13 out of 18 patients were in stage 1, 4 were in stage 2, and 1 was in stage 3. In the acromegaly cohort, 11 of 12 patients were stage 1 and one was stage 2. Of the 13 patients in the NFPA group, 12 were stage 1 and 1 patient was stage 2. Highly correlated variables (FBG–HbA1c, FBG–HOMA, LDL–Total cholesterol, Insulin–HOMA) were excluded, and a multivariable logistic regression model was built including age, BMI, LDL, HbA1c, LVEDD, LVPW thickness, RV diameter, and diagnostic group. The independent predictors of LVDD in multivariable logistic regression were age (OR = 1.265, 95% CI: 1.155–1.385, *p* < 0.001) and diagnostic group (overall *p* = 0.004). Compared with the Control group, the risk of LVDD was significantly higher in patients with Prolactinoma (OR = 10.9, 95% CI: 1.344–89.676, *p* = 0.025), Acromegaly (OR = 167.5, 95% CI: 10.3–2721.4, *p* < 0.001), and NFPA (OR = 12.0, 95% CI: 1.4–104.8, *p* = 0.024). LVPW thickness showed a positive trend toward association with LVDD, though it did not reach statistical significance (OR = 1.946, 95% CI: 0.931–4.068, *p* = 0.077). Other clinical and echocardiographic variables, including BMI, LDL, HbA1c, LVEDD, and RV diameter, were not independently associated with LVDD (all *p* > 0.05) (Table 3 and Table 4). In addition, the rates of diastolic dysfunction observed in the study groups are shown in Figure 1.

## 4. Discussion

This study compared cardiometabolic characteristics, cardiac structure, and left ventricular diastolic function among patients with acromegaly, prolactinoma, NFPA, and apparently healthy controls. LVDD was present in 21.5% of the overall cohort and was most frequent in acromegaly (46.2%), followed by NFPA (28.9%), prolactinoma (19.8%), and controls (5.8%). Most cases were grade I, while advanced dysfunction was uncommon. Age and diagnostic group remained independently associated with LVDD; however, the cross-sectional design, small number of events in some subgroups, and wide confidence intervals warrant cautious interpretation.

Acromegaly showed the most consistent pattern of diastolic impairment, with lower mitral E-wave velocity, E/A ratio, and lateral and average e′ velocities, together with higher LAVI. The average E/e′ ratio was also highest in this group. Nevertheless, average E/e′ values in all groups remained below the conventional threshold of >14, and TRV did not differ significantly. These findings therefore suggest predominantly impaired myocardial relaxation rather than a uniform elevation in left ventricular filling pressure. The average E/e′ values in prolactinoma and NFPA were comparable to that in controls, indicating less pronounced diastolic involvement.

Cardiac manifestations of acromegaly are heterogeneous and may vary according to age, disease duration, biochemical activity, cardiometabolic comorbidities, and treatment exposure. Previously reported abnormalities include ventricular wall thickening, altered geometry, impaired relaxation, and less consistently, systolic dysfunction [12]. In a meta-analysis of seven studies comprising 458 untreated patients, Guo et al. reported a pooled LVDD prevalence of 50.5% [13], which was close to the 46.2% observed in our acromegaly group. Lower rates have been described in more heterogeneous cohorts. Akdeniz et al. reported LVDD in 35.7% of patients with active, controlled, or cured acromegaly, together with an increased septal E/Em ratio despite preserved LVEF [14]. Other studies involving different disease stages have documented less marked diastolic involvement [15], including prevalence rates of approximately 15.8% and 11.3% reported by Hinojosa-Amaya et al. [16] and Cansu et al. [17], respectively. Differences in patient selection, disease activity, treatment history, comorbidity burden, and echocardiographic definitions may account for this variation.

Earlier Doppler studies provide further context for our findings. Leães et al. demonstrated lower transmitral E/A and tissue Doppler early-to-late diastolic velocity ratios in acromegaly, with an E/A ratio < 1 in 41% of patients by conventional Doppler and in approximately half by tissue Doppler [18]. Mercuro et al. reported prolonged global and regional IVRT and inversion of regional early-to-late diastolic velocity ratios despite preserved LVEF [19]. Galderisi et al. similarly identified delayed myocardial relaxation and reduced diastolic tissue velocity ratios at the septal, mitral annular, and tricuspid sites in active acromegaly without global systolic impairment [20]. Di Bello et al. found a lower E/A ratio and reduced early diastolic strain rate in untreated active disease, together with regional systolic deformation abnormalities despite preserved LVEF [21]. Collectively, these observations are consistent with the lower relaxation velocities and prolonged lateral IVRT in our acromegaly group and support an early functional phenotype rather than overt systolic dysfunction. The predominance of grade I LVDD suggests that echocardiography may help identify early relaxation abnormalities, particularly in patients with acromegaly and additional cardiometabolic risk factors. However, given the cross-sectional design and the absence of outcome data, our findings neither support universal echocardiographic screening nor justify specific treatment decisions based solely on these abnormalities.

The clinical and metabolic profile of our acromegaly cohort may also have contributed to the observed diastolic abnormalities. Although the group was relatively young, the cross-sectional design did not allow the relative contributions of hormone excess, metabolic factors, and other disease-related characteristics to be distinguished. Our patients with acromegaly had the highest BMI, waist circumference, fasting glucose, HbA1c, insulin, and HOMA-IR values, consistent with evidence that cardiac abnormalities in acromegaly often coexist with adverse metabolic and body-composition profiles [22]. Although HbA1c was higher in the acromegaly group, it was not independently associated with LVDD in the multivariable analysis. Nevertheless, an unfavorable glycemic profile may contribute to adverse myocardial and cardiovascular changes and therefore remains clinically relevant in the overall cardiovascular risk assessment of patients with acromegaly [4]. In this context, Akdeniz et al. identified age and diabetes, rather than disease activity or duration, as independent predictors of LVDD [14]. Direct comparison with their findings should be cautious, however, because patients with diabetes mellitus were excluded from our cohort. Thus, the observed changes may reflect the combined influence of GH/IGF-1 exposure, obesity, insulin resistance, age, and other cardiovascular factors. Although HbA1c was not independently associated with LVDD, this does not exclude a metabolic contribution, given the subgroup size and intercorrelations among glycemic variables. LVEF was lower than in controls but remained within the preserved range, and modest differences in LVESD, posterior-wall thickness, aortic diameter, and right atrial diameter were insufficient to establish a distinct cardiomyopathic phenotype.

Some cardiac abnormalities in acromegaly may be modifiable. A recent systematic review described improvements in several structural and functional indices following the first GH-lowering intervention, although study designs, treatments, follow-up periods, and outcome definitions differed [23]. Wolf et al. likewise suggested that reducing GH and IGF-1 exposure may improve ventricular mass and diastolic indices, whereas established myocardial or valvular abnormalities may persist [24]. Because our study did not evaluate longitudinal changes or stratify patients by biochemical control and treatment modality, it cannot determine whether the observed findings preceded treatment, persisted despite hormonal control, or might improve with further therapy.

Evidence regarding cardiac involvement in prolactinoma is more limited. LVDD was identified in 19.8% of our prolactinoma group, predominantly as grade I dysfunction. Lateral e′ velocity was lower than in controls, whereas LAVI and average E/e′ were comparable, suggesting mild impairment of relaxation without consistent evidence of elevated filling pressure.

Arcopinto et al. reported prolonged IVRT, increased atrial filling velocity, and overt diastolic dysfunction in approximately 16% of 24 women with chronic hyperprolactinemia [7]. This proportion is close to that observed in our cohort, although differences in sex distribution, treatment status, disease duration, and diagnostic criteria limit direct comparison. Jiang et al. detected impaired left ventricular longitudinal function by speckle-tracking echocardiography despite relatively preserved conventional measurements [6]. Because myocardial deformation imaging was unavailable in our study, such subclinical systolic changes could not be evaluated. Conversely, Yazici et al. found no differences in conventional systolic or diastolic function between 67 patients with prolactinoma and 57 controls, although CIMT was greater in the prolactinoma group [25]. These findings suggest that myocardial and vascular abnormalities are not consistently observed across prolactinoma cohorts or necessarily present in parallel.

The prolactinoma group also had higher insulin and HOMA-IR values than controls and the highest LDL concentration. These metabolic differences may influence cardiovascular risk independently of prolactin exposure. A systematic review and meta-analysis reported improvements in weight, lipid profile, and glucose regulation after dopamine-agonist therapy, but the contributing studies were heterogeneous and largely observational [26]. In our cohort, information on dopamine-agonist type and cumulative exposure, biochemical control, disease duration, and gonadal status was not consistently available. The higher LVDD prevalence therefore cannot be attributed specifically to prolactin excess, and contributions from hypogonadism, metabolic abnormalities, treatment, and residual confounding remain possible.

The 28.9% prevalence of LVDD in NFPA was higher than that observed in the prolactinoma and control groups despite the absence of hormone hypersecretion. The NFPA group also had a lower E/A ratio than controls, whereas average E/e′, LAVI, and TRV were comparable, and most cases were grade I. This pattern again favors impaired relaxation over consistently elevated filling pressure.

Direct evidence concerning NFPA and LVDD appears to be lacking. Çağır et al. reported increased arterial stiffness in NFPA, and a meta-analysis by Bioletto et al. found excess mortality compared with the general population; however, neither study directly assessed left ventricular diastolic function [27,28]. Hypopituitarism may represent one possible contributor. Özbey et al. demonstrated prolonged DT and IVRT in adults with GH-deficient hypopituitarism despite preserved systolic function [29]. Pituitary insufficiency was more common in our NFPA group, but individual axis deficiencies and replacement adequacy were not evaluated. Moreover, the Özbey cohort was not restricted to NFPA and included patients with multiple hormone deficiencies. The association observed in our study should therefore remain hypothesis-generating pending prospective evaluation in well-characterized NFPA populations.

CIMT differed across the groups, but the only significant pairwise contrast was between NFPA and prolactinoma; NFPA did not differ from controls. Cansu et al. reported higher CIMT in acromegaly than in healthy individuals, a finding not reproduced in our cohort, possibly because of differences in disease activity, treatment, or cardiometabolic characteristics [17]. Jiang et al. found increased CIMT and an independent positive association with prolactin levels in untreated prolactinoma, whereas our prolactinoma group did not show a similar pattern; incomplete information on treatment and biochemical control limits comparison [30]. NFPA-specific CIMT data remain scarce. Although Çağır et al. identified increased arterial stiffness in NFPA, this measure cannot be equated with carotid wall thickening [27]. Consequently, the higher CIMT in NFPA than in prolactinoma should be regarded as an isolated relative difference rather than evidence of generalized subclinical atherosclerosis. MPV was lower in acromegaly, but its clinical significance remains uncertain without platelet-function measurements or prospective vascular outcomes.

Age was independently associated with LVDD, with an odds ratio of 1.265 per year, consistent with the established effect of aging on ventricular relaxation and previous findings in acromegaly [14]. Diagnostic group also remained significant after adjustment for BMI, LDL-C, HbA1c, LVEDD, posterior-wall thickness, and right ventricular diameter. Nevertheless, the group-specific estimates had wide confidence intervals, particularly for acromegaly, and only three controls had LVDD. The model therefore supports an association between diagnostic category and LVDD but neither provides precise effect estimates nor establishes causality.

Overall, the cardiovascular phenotype appeared to differ among pituitary adenoma subtypes. Acromegaly showed the clearest combination of impaired relaxation, greater LAVI, and LVDD, whereas prolactinoma displayed more limited relaxation-related and metabolic changes. NFPA had a comparatively high LVDD prevalence despite lacking hormone hypersecretion. However, the predominance of grade I dysfunction, E/e′ values below 14, and the absence of concordant abnormalities across all diastolic indices argue against advanced diastolic disease or uniformly elevated filling pressure.

Clinically, these findings support careful evaluation of conventional cardiovascular risk factors, including age, adiposity, glucose metabolism, blood pressure, and lipid status. Echocardiography may be considered in patients with cardiovascular symptoms, multiple risk factors, longstanding or active hormonal disease, or suspected cardiac involvement. However, this study did not assess the clinical benefit of routine echocardiographic screening and cannot support universal screening of all patients with pituitary adenomas.

The study has several limitations. The cross-sectional, single-center design prevents conclusions regarding causality, temporal progression, or reversibility. Group sizes were unequal, the acromegaly subgroup was relatively small, and the limited number of LVDD events—particularly among controls—reduced the precision of the multivariable estimates. No formal a priori sample-size calculation was performed.

Detailed information on disease duration, biochemical activity and control, and previous or ongoing disease-specific treatment was not consistently available across the three patient groups. Hormone-replacement therapy data were also incomplete and were therefore excluded from the analyses. Although pituitary surgical history and the presence of pituitary insufficiency were reported descriptively, these variables do not establish biochemical control or the adequacy of hormone replacement at the time of echocardiography. This limitation is particularly relevant in acromegaly, where cardiac abnormalities may vary according to disease activity and treatment response. Consequently, the observed between-group differences cannot be attributed solely to the underlying adenoma subtype or hormone excess, because the contributions of disease activity, previous treatment, and pituitary hormone deficiencies could not be distinguished. These findings should therefore be interpreted as associations with diagnostic groups in a clinically heterogeneous population.

Cardiac assessment relied primarily on conventional and tissue Doppler echocardiography; speckle-tracking strain, three-dimensional echocardiography, cardiac magnetic resonance imaging, and circulating cardiac biomarkers were unavailable. Formal intraobserver and interobserver reproducibility was not assessed, and the number and management of indeterminate diastolic-function examinations were not explicitly documented. TSH- and ACTH-secreting pituitary adenomas were not included because their numbers were insufficient for meaningful subgroup analyses, limiting generalizability to these less common functional subtypes. Finally, the large number of clinical, metabolic, and echocardiographic comparisons increases the possibility of chance findings; isolated differences without corresponding changes in related measures should therefore be considered exploratory.

## 5. Conclusions

In conclusion, LVDD was more frequent in all three pituitary adenoma groups than in controls and was most prevalent in acromegaly. Acromegaly also demonstrated the most coherent pattern of impaired relaxation, with lower mitral E-wave velocity, E/A ratio, and lateral and average e′ velocities, together with greater LAVI and the highest average E/e′. Nevertheless, group median E/e′ values remained below 14, most LVDD cases were grade I (84.8%), and LVEF was preserved. These findings indicate predominantly impaired relaxation, with advanced diastolic dysfunction being uncommon in this cohort. The mechanisms underlying the higher LVDD prevalence in prolactinoma and NFPA remain uncertain. Larger prospective studies incorporating disease activity, cumulative hormone exposure, treatment status, pituitary-axis function, advanced cardiac imaging, and longitudinal outcomes are needed to determine the clinical significance and reversibility of these findings.

## Figures and Tables

**Figure 1:**
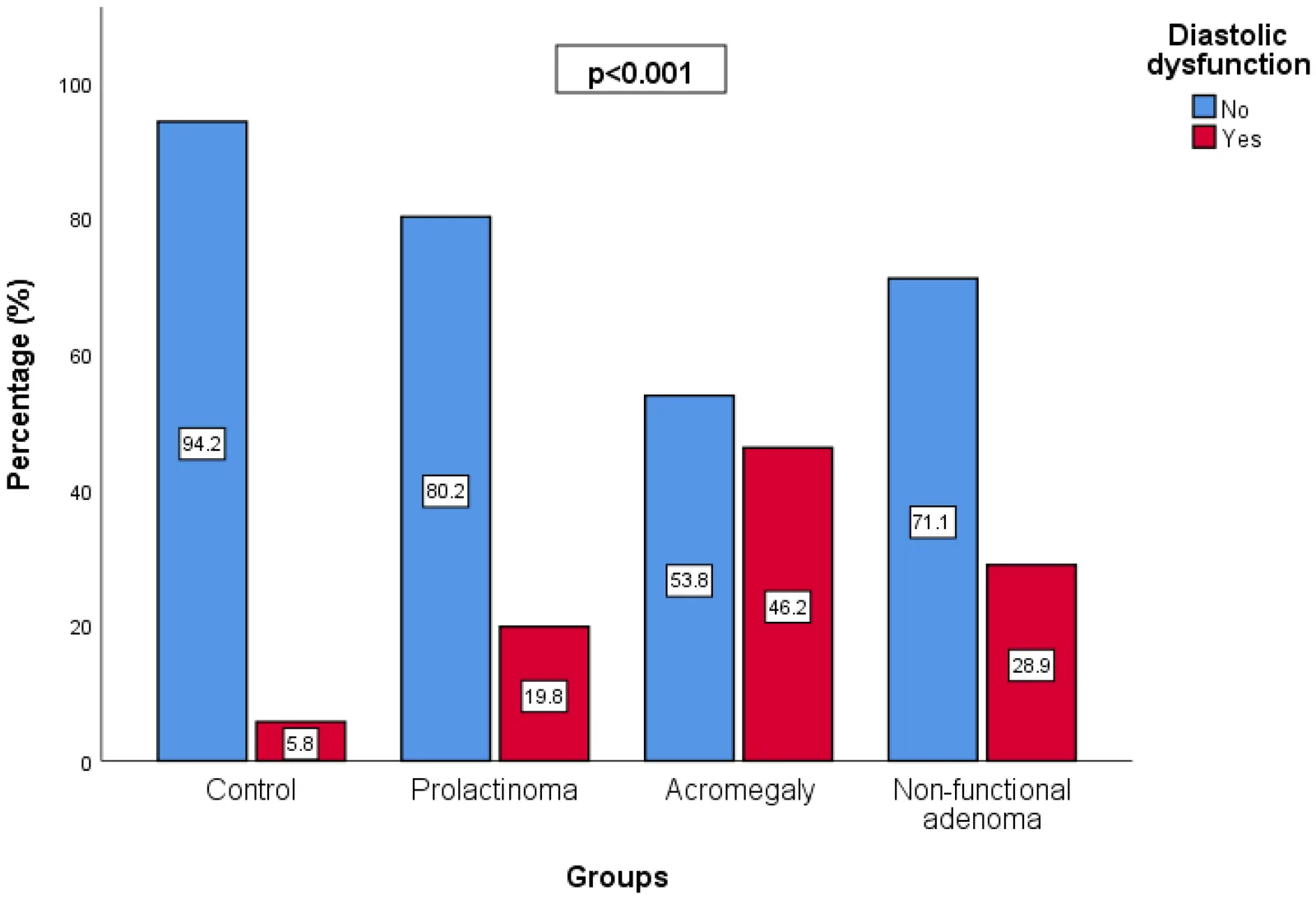
Diastolic dysfunction rates in study groups.

**Table 1:** Clinical and demographic characteristics of the study population.

		Groups				*p*
		Control	Prolactinoma	Acromegaly	NFPA	
**Age (years)**		43.7 ± 12.1	39.4 ± 11.4	38.2 ± 8.7	42.5 ± 16.1	0.120
**Height (cm)**		163.9 ± 7.0	165.4 ± 7.7	167.9 ± 5.6	169.3 ± 7.9	0.001
**Weight (kg)**		70.0 ± 12.7	78.1 ± 15.8	88.7 ± 14.3	80.9 ± 14.8	<0.001
**BMI (kg/m^2^)**		25.9 ± 3.6	28.6 ± 5.9	31.0 ± 5.1	28.4 ± 5.4	0.001
**Waist circumference (cm)**		88.8 ± 12.1	100.7 ± 12.3	107.2 ± 7.2	101.9 ± 13.7	<0.001
**Sex**	M	20 (38.5)	26 (28.6)	14 (53.8)	16 (35.6)	0.115
	F	32 (61.5)	65 (71.4)	12 (46.2)	29 (64.4)	
**Surgical history**	No	-	67 (73.6)	4 (15.4)	21 (46.7)	<0.001
	Yes	-	24 (26.4)	22 (84.6)	24 (53.3)	
**Pituitary insufficiency**	No	-	73 (80.2)	20 (76.9)	26 (57.8)	0.019
	Yes	-	18 (19.8)	6 (23.1)	19 (42.2)	
**Tumor size (mm)**		-	6 (3–10)	10 (7–12)	9 (5–20.2)	0.013

NFPA: Nonfunctioning pituitary adenoma, BMI: Body Mass Index. Results were presented as mean ± Standard Deviation, or n (%), or median (IQR). Surgical history and pituitary insufficiency were compared only among the patient groups.

**Table 2:** Comparison of biochemical and metabolic parameters among the study groups.

	Groups				*p*
	Control	Prolactinoma	Acromegaly	NFPA	
**Fasting Blood Glucose (mg/dL)**	94 (87–98)	95.5 (88–106)	107 (103–122)	94 (88–103)	**<0.001**
**HbA1c (%)**	5.2 (5–5.6)	5.5 (5.2–5.8)	5.8 (5.4–5.9)	5.5 (5.2–5.8)	**<0.001**
**LDL (mg/dL)**	123 (100–150)	140.5 (116–170)	116 (105–148)	120 (105–154)	**0.003**
**HDL (mg/dL)**	37 (32–44)	38 (33–45)	37 (33–43)	44 (35–51)	0.307
**Total Cholesterol (mg/dL)**	184.5 (167–229.5)	202 (173–239)	185 (163–196)	197 (169–238)	0.118
**Triglycerides (mg/dL)**	117 (78.5–183.5)	144 (91–231)	129 (116–223)	160 (136–220)	0.051
**Insulin (mIU/L)**	6 (4–7)	11 (6–17)	14 (13–22)	8.8 (4.8–11.2)	**<0.001**
**HOMA-IR**	1.4 (0.9–1.7)	2.6 (1.5–4.1)	3.6 (3.4–7.9)	2.2 (1–3)	**<0.001**
**MPV (fL)**	10.5 (9.8–10.9)	9.4 (8.2–10.5)	7.9 (7.8–9.1)	9.9 (8.8–10.8)	**<0.001**

NFPA: Nonfunctioning pituitary adenoma, HbA1c: glycated hemoglobin, LDL: Low-density Lipoprotein, HDL: High-Density Lipoprotein, HOMA-IR: Homeostasis Model Assessment for Insulin Resistance, MPV: Mean Platelet Volume. Results were presented as median (IQR).

**Table 3:** Comparison of echocardiographic parameters among the study groups.

	Groups				*p*
	Control	Prolactinoma	Acromegaly	NFPA	
**Aortic diameter (mm)**	27 (25–30)	29 (26–31)	29 (27–34)	30 (27–31)	**0.033**
**LA diameter (mm)**	33 (30–36)	35 (32–38)	35 (33–37)	35 (33–37)	0.100
**IVS thickness (mm)**	9 (9–10)	9 (8–10)	10 (9–10)	9 (8–10)	0.107
**LVEDD (mm)**	46 (44–48)	46 (44–49)	47 (45–55)	49 (46–50)	**0.010**
**LVESD (mm)**	27 (25–29)	28 (26–31)	29 (27–35)	28 (27–33)	**0.001**
**LVPW thickness (mm)**	9 (9–10)	9 (9–10)	10 (9–10)	9 (8–10)	**0.026**
**LVEF (%)**	69 (66–72)	68 (65–70)	65 (62–69)	68 (65–70)	**0.004**
**RA diameter (mm)**	30 (28–32)	30 (27–32)	30 (28–36)	29 (27–30)	**0.006**
**RV diameter (mm)**	22 (20–24)	23 (20–25)	24 (22–30)	24 (21–25)	0.062
**Mitral E-wave velocity (cm/s)**	83 (71–97)	84 (71–90)	71 (57–78)	79 (68–86)	**<0.001**
**Mitral A-wave velocity (cm/s)**	65 (59–78)	67 (59–79)	73 (62–85)	67 (58–84)	0.252
**E/A ratio**	1.3 (1.2–1.4)	1.2 (0.9–1.4)	0.8 (0.8–1.2)	1.1 (0.8–1.4)	**<0.001**
**DT (ms)**	179 (166–193)	181 (167–210)	186 (162–219)	171 (167–189)	0.480
**Tricuspid E-wave velocity (cm/s)**	52 (47.5–61)	56 (47–65)	54 (47–57)	52 (44–60)	0.185
**Tricuspid A-wave velocity (cm/s)**	44 (36.5–51.5)	49 (40–60)	43 (39–43)	48 (43–52)	**0.026**
**TAPSE (mm)**	23 (22–26)	24 (22–27)	23 (20–24)	24 (22–25)	0.572
**Lateral e’ velocity (cm/s)**	14 (13–15)	13 (11–14)	8 (7–10)	12 (9–15)	**<0.001**
**Lateral a’ velocity (cm/s)**	8 (6–10.5)	9 (8–11)	10 (7–11)	10 (9–12)	**0.005**
**Lateral s’ velocity (cm/s)**	8 (7–9)	9 (7–11)	8 (8–11)	8 (7–10)	0.090
**Lateral IVRT (ms)**	74 (61–89)	72 (55–83)	105 (67–161)	78 (67–91)	**0.008**
**Lateral IVCT (ms)**	82 (67–107)	83 (67–100)	67 (50–72)	78 (61–94)	**0.017**
**Lateral ET (ms)**	277 (258–295)	261 (227–291)	261 (233–283)	261 (238–294)	0.083
**Septal e’ velocity (cm/s)**	10 (9–11)	10 (8–13)	8 (6–10)	10 (7–13)	0.053
**Septal a’ velocity (cm/s)**	7 (6–9)	9 (7–10)	10 (8–12)	9 (7–11)	**<0.001**
**Septal s’ velocity (cm/s)**	7 (5.5–8)	8 (6–9)	8 (6–10)	8 (6–9)	**0.025**
**Septal IVRT (ms)**	85 (69.5–106.5)	72 (61–94)	78 (67–105)	83 (61–94)	**0.032**
**Septal IVCT (ms)**	70.5 (57.5–89.5)	72 (55–89)	50 (39–78)	78 (61–89)	**0.026**
**Septal ET (ms)**	285.5 (255–305)	266 (240–294)	283 (233–305)	272 (250–294)	0.056
**Average E/e’ ratio**	6.4 (5.2–7.2)	6.4 (5.7–7.6)	7.5 (7.1–8.7)	6.4 (5.6–8.0)	**0.001**
**Average e′ velocity (cm/s)**	12 (11–13)	11.5 (9.5–14)	7.8 (7–10.5)	11.5 (7.5–14)	**<0.001**
**RV e’ velocity (cm/s)**	11 (9–14)	12 (10–16)	11 (10–13)	12 (10–16)	0.084
**RV a’ velocity (cm/s)**	14 (10.5–16.5)	14 (12–16)	16 (13–18)	16 (12–19)	0.072
**RV s’ velocity (cm/s)**	11 (10–13)	11 (9–13)	10 (10–13)	12 (10–13)	0.635
**RV IVRT (ms)**	76 (57–92)	61 (50–89)	61 (50–81)	67 (50–89)	0.104
**RV IVCT (ms)**	55 (44–77.5)	61 (50–78)	72 (50–72)	55 (44–72)	0.294
**RV ET (ms)**	274.5 (251–299)	266 (244–294)	255 (227–289)	272 (250–299)	0.143
**LAVI (mL/m^2^)**	28.5 (25.5–31.8)	29.9 (27.5–31.9)	30.7 (30.0–32.3)	29.4 (27.5–31.9)	**0.017**
**TRV (m/s)**	2.3 (2.1–2.6)	2.3 (2.1–2.5)	2.4 (2.3–2.6)	2.3 (2.1–2.5)	0.157
**CIMT (mm)**	0.6 (0.5–0.6)	0.5 (0.5–0.6)	0.6 (0.4–0.8)	0.6 (0.5–0.7)	**0.026**

NFPA: Nonfunctioning pituitary adenoma, LA: Left atrium, IVS: Interventricular septum, LVEDD: Left ventricular end-diastolic diameter, LVESD: Left ventricular end-systolic diameter, LVPW: Left ventricular posterior wall, LVEF: Left ventricular ejection fraction, RA: Right atrium, RV: Right ventricle, DT: E-wave deceleration time, IVRT: Isovolumetric relaxation time, IVCT: Isovolumetric contraction time, ET: Ejection Time, TAPSE: Tricuspid Annular Plane Systolic Excursion, LAVI: Left atrial volume index, TRV: Tricuspite regurgitation velocity, CIMT: Carotid intima-media thickness. Results were presented as median (IQR).

**Table 4:** Factors associated with left ventricular diastolic dysfunction in multivariable logistic regression analysis.

Variable	OR (95% CI)	*p*-Value
**Age (years)**	1.265 (1.155–1.385)	**<0.001**
**BMI (kg/m^2^)**	0.999 (0.898–1.110)	0.979
**LDL (mg/dL)**	1.005 (0.990–1.020)	0.515
**HbA1c (%)**	0.921 (0.239–3.554)	0.904
**LVEDD (mm)**	1.005 (0.888–1.290)	0.949
**LVPW thickness (mm)**	1.946 (0.931–4.068)	0.077
**RV diameter (mm)**	1.070 (0.888–1.290)	0.476
**Control**	**Reference**	**0.004**
**Prolactinoma**	10.9 (1.344–89.676)	**0.025**
**Acromegaly**	167.5 (10.3–2721.4)	**<0.001**
**NFPA**	12.0 (1.4–104.8)	**0.024**

BMI: Body mass index, LDL: Low density lipoprotein, HbA1c: Glycated hemoglobin, LVEDD: Left ventricular end-diastolic diameter, LVPW: Left ventricular posterior wall, RV: Right ventricle, NFPA: Nonfunctioning pituitary adenoma.

## Data Availability

The data presented in this study are available on request from the corresponding author due to privacy and ethical restrictions. The data are not publicly available to protect patient confidentiality.
